# Mineral Trioxide Aggregate—A Review of Properties and Testing Methodologies

**DOI:** 10.3390/ma10111261

**Published:** 2017-11-02

**Authors:** William N. Ha, Timothy Nicholson, Bill Kahler, Laurence J. Walsh

**Affiliations:** 1 School of Dentistry, University of Queensland, Herston, Brisbane 4004, Australia; wyattkahler@bigpond.com (B.K.); l.walsh@uq.edu.au (L.J.W.); 2 School of Chemical Engineering, University of Queensland, St. Lucia, Brisbane 4067, Australia; t.m.nicholson@uq.edu.au

**Keywords:** hygroscopic dental cement, bioceramic, endodontics, dental materials, biocompatibility, physical properties, setting time, rheology, calcium silicate cement, solubility

## Abstract

Mineral trioxide aggregate (MTA) restoratives and MTA sealers are commonly used in endodontics. Commonly referenced standards for testing of MTA are ISO 6876, 9917-1 and 10993. A PubMed search was performed relating to the relevant tests within each ISO and “mineral trioxide aggregate”. MTA restoratives are typically tested with a mixture of tests from multiple standards. As the setting of MTA is dependent upon hydration, the results of various MTA restoratives and sealers are dependent upon the curing methodology. This includes physical properties after mixing, physical properties after setting and biocompatibility. The tests of flow, film thickness, working time and setting time can be superseded by rheology as it details how MTA hydrates. Physical property tests should replicate physiological conditions, i.e., 37 °C and submerged in physiological solution. Biocompatibility tests should involve immediate placement of samples immediately after mixing rather than being cured prior to placement as this does not replicate clinical usage. Biocompatibility tests should seek to replicate physiological conditions with MTA tested immediately after mixing.

## 1. Introduction

Mineral Trioxide Aggregate (MTA) was first described in 1993 as a cement used for its use in repairing lateral root perforations [1]. Its composition was described as being primarily a mixture of calcium silicates comprised of calcium oxide (CaO) (50–75% *w*/*w*) and silicon dioxide (SiO_2_) (15–25% *w*/*w*) [2]. Calcium silicates are not particularly radiopaque, and thus a radiopaque agent such as bismuth oxide was then added [2]. Since its invention, MTA has been tested under laboratory conditions, then in animal studies and in clinical trials [3,4,5]. The positive results of these investigations have resulted in MTA becoming a commonly used material in pediatric dentistry and in endodontics [6,7].

### 1.1. Terminology

As well as the term MTA, other words have been used to describe these types of materials. The term “bioceramics”, which was originally used for a material known as BioAggregate^®^ (Innovative Bioceramix, Vancouver, BC, Canada) has been used for MTA-like cements [8]. Despite the differences in terminology, these cements are similar in their elemental compositions [9,10,11]. This is illustrated in Table 1. BioAggregate contains 38.5% calcium, 11.5% silicon and 10.6% tantalum [11]. When converted to their oxide forms using element-to-stoichiometric oxide conversion, the respective weights of calcium oxide and silicon dioxide would be 53.9% and 24.60%. If tantalum was removed in its oxide form from the whole sample (i.e., 12.9% of Ta_2_O_5_, leaving 87.1%), the remaining percentages would be 61.8% CaO and 28.3% SiO2. These values place the material well within the definition of MTA.

Another product that is not marketed as MTA but is chemically similar is Biodentine^TM^ (Septodont, Saint-Maur-des-Fossés, France). The elemental composition is 46.3% calcium, 9.8% silicon and 2.7% zirconium. If a similar element to stoichiometric oxide conversion is performed, the remaining cement percentages once the radiopacifier is removed are 67.2% CaO and 21.8% SiO_2_, values which again place the material within the definition of MTA.

If one defines bioceramics as “non-metallic inorganic materials” [12], then this encompasses the powdered components of MTA, as well as zinc phosphate, zinc oxide eugenol and glass ionomer dental cements, materials which do not share many similarities at the chemical level. Hence, this definition of bioceramics has little functional purpose and should not be used. Nevertheless, products which claim to be either MTA or bioceramic sealers have appeared.

Table 2 illustrates the compositions of popular MTA or bioceramic sealers. BioRoot^TM^ RCS (Septodont, St. Maure de Fosses, France) is the most MTA-like of the sealers as it involves a powder mixed with water. MTA Fillapex^®^ (Angelus, Londrina, Brazil) is predominately salicylate resin, but with some MTA included as an additive and is therefore not primarily an MTA cement [13]. Likewise, iRoot^®^ SP (Innovative BioCeramix, Vancouver, BC, Canada) and EndoSeal MTA (Maruchi, Wonju-si, Korea) are single-component injectable pastes that do not contain any water but use a thickening agent to create a gel-like paste [14]. The lack of water for setting reaction puts this material outside the definition of MTA.

### 1.2. Performance Testing

A range of techniques have been used to assess the performance of MTA. The purpose of this paper is to explore the published literature on the testing of MTA and therefore identify key aspects of the testing methodologies used, what insights they reveal as to the behavior of the material, and what the limitations are of widely used international testing standards and how these relate to clinical performance. Because the performance of MTA is affected by the conditions used in the testing environment, significant concerns arise when standardized testing does not represent physiological or clinical conditions.

Typical international standards (ISO) that have been used to assess the properties of MTA comprise:

- ISO 6876, which tests the physical properties of endodontic sealers [16];
- ISO 9917-1, which tests the physical properties of restorative cements [17]; and
- ISO 10993 which tests the biocompatibility of medical devices [18].

### 1.3. Aims

This review aims to:

- Describe the commonly used ISO tests for MTA;
- List findings from the literature on MTA using these tests;
- Identify problems with the methods used for testing MTA; and
- Suggest alternative testing methods.

## 2. Materials and Methods

A PubMed search was undertaken using “Mineral Trioxide Aggregate” combined with the following terms:

From testing methods for ISO 6876:

- flow;
- working time;
- setting time;
- film thickness;
- dimensional change [19], which has been removed in the latest version;
- solubility;
- and radiopacity [16].

From testing methods for ISO 9917-1:

- setting time;
- compressive strength;
- acid erosion;
- acid-soluble arsenic and lead contents;
- and radiopacity [17].

From testing methods for ISO 10993:

- genotoxicity, carcinogenicity and reproductive toxicity (ISO 10993-3) [20];
- cytotoxicity (ISO 10993-5) [21]; and local effects after implantation (ISO 10993-6) [22].

From the results, when multiple studies were found, studies which compared MTA products against Super EBA (Harry J Bosworth Co., Skokie, IL, USA), glass ionomer cement, “bioceramics” or AH Plus^®^ (Dentsply DeTrey, Konstanz, Germany) were prioritized. This was done to enable meaningful comparison of MTA against its contemporary alternatives.

The results of each search term were reviewed, and the methodologies were considered in light of the known properties of MTA. These properties were grouped as follows:

Properties after mixing:

- flow (ISO 6876);
- working time (ISO 6876);
- setting time (ISO 6876 and 9917-1);
- and film thickness (ISO 6876).

Properties after setting:

- dimensional change (ISO 6876);
- solubility (ISO 6876); and
- radiopacity (ISO 6876 and 9917-1);
- compressive strength (ISO 9917-1);
- acid erosion (ISO 9917-1);
- acid-soluble arsenic and lead contents (ISO 9917-1);
- genotoxicity, carcinogenicity and reproductive toxicity (ISO 10993-3);
- cytotoxicity (ISO 10993-5); and
- local effects after implantation (ISO 10993-6).

## 3. Results

Many of the materials that have been tested using ISO 6876 for endodontic sealers have been indicated for use as an endodontic sealer. These products are henceforth called “MTA sealers”, irrespective of whether the material is marketed as an MTA or as a bioceramic. Similarly, many materials that have been tested using ISO 9917.1 for restorative cements are described as “MTA restoratives”, irrespective of whether the material is marketed as an MTA or a bioceramic.

### 3.1. Properties After Mixing

The results of tests on MTA restoratives and MTA sealers involving properties after mixing are summarized in Table 3.

#### 3.1.1. Flow (ISO 6876)

This test involves placing sealer on the center of a glass plate. After waiting for 180 s, a glass plate is placed on top of the dispensed sealer. After 10 min, the diameter of the sealer is to be measured. If the diameter is less than 17 mm, the material does not comply with the standard [16].

#### 3.1.2. Film Thickness (ISO 6876)

Sealer is placed on a glass plate. After 180 s, another glass plate is placed on top of the sealer, with a load of 150 N [16]. The load needs to compress the sealer such that it completely fills the area between the glass plates. After 10 min, the distance between the two plates is determined, to measure the thickness of the film of sealer [16].

#### 3.1.3. Working Time (ISO 6876)

This test is similar to the flow test ISO 6876. Instead of delaying the compression by the glass plates by 180 s, the cement is tested at longer time points, until the specimen diameter is 10% less than the tested diameter at 180 s [16].

#### 3.1.4. Setting Time (ISO 6876 and 9917-1)

For MTA restoratives, cements are mixed and placed into a mold. An indentation needle with a diameter of 1 mm and a mass of 400 g is placed gently onto the setting cement at progressive time points. If a full circular indentation appears upon placement of the needle, the cement is unset. If the indentation is incomplete, it is deemed as set [17].

For MTA sealers, sealers are placed into ring-shaped molds. Sealers that require moisture to set are placed into a dental plaster mold. The mold is pre-treated by storing it in 95% humidity for 24 h prior to placing the sealer. Sealers that do not require moisture to set are placed into a metal mold [16].

Of note, the American National Standards Institute’s American Dental Association standards for initial and final setting times resemble the ISO 6876 values for initial setting time and ISO 9917.1 for final setting time [16,17,37].

### 3.2. Properties After Setting

The results of non-biological tests on MTA restoratives and MTA sealers involving properties after setting are summarized in Table 4. The results for acid erosion and acid-soluble arsenic and lead contents are not included in Table 4 as the studies are few and results are highly variable. The results of non-biological results are not summarized in a table like Table 4 because biological tests do not standardize precise cell lines, location for implantation or animals tested.

#### 3.2.1. Dimensional change (ISO 6876)

Materials are mixed and placed in polyethylene molds. Once set, they are removed from the mold and the length measured. After storage in distilled water for 30 days at room temperature, the length is re-measured. To conform with the standard, samples should not exceed 0.1% in shrinkage or 0.1% in expansion [19].

Samples that require moisture to set are mixed with 0.02 mL of water per 2 g of material prior to placement into the mold [19]. This is a ratio of 0.01 g water: 1 g powder, which does not align with the manufacturer’s recommended ratio of 0.33 g water to 1 g powder. Therefore, MTA cements that are placed into the mold without mixing water will be too dry. To overcome this problem of inadequate water, in one study iRoot SP was tested by being held between two pieces of wet cloth, located between the mold and the glass plates, prior to immersing the mold into water [35].

There are no known published results using the ISO 6876 test for dimensional change for MTA restoratives.

As iRoot SP is highly soluble [35], the results for dimensional change are difficult to interpret in terms of what may have happened if the cement was not given added water prior to placement in the mold.

#### 3.2.2. Solubility (ISO 6876)

Solubility tests for MTA are performed by placing set samples into distilled water for 24 h to room temperature. Any residue that enters the water is then measured. To conform to the standard, sealers should not be more soluble by more than 3% by mass [16].

#### 3.2.3. Radiopacity (ISO 6876 and 9917-1)

A 1 mm thick sample of MTA placed beside an aluminum step wedge of steps of 0.5 or 1 mm is exposed to X-rays at 65 kV. The radiopacity of the sample is compared to the step wedge, and the equivalent mm thickness of aluminum (mm Al) determined [16,17].

ISO 6876 specifies that sealers must be a minimum of 3 mm Al [16]. ISO 9917:2007 requires samples to be stored for no more than seven days before testing [17].

#### 3.2.4. Compressive Strength (ISO 9917)

This test involves placing samples within molds for only one hour prior to testing [17]. However, as MTA cements take longer than one hour to set, some consider curing MTA for 24 h [17,50].

This test does not have a method (such as a gypsum mold) which requires diffused ambient water to aid in the setting reaction [17]. Therefore, MTA cements that lack mixing water need the intentional addition of water, or otherwise they will not solidify and hence will have no compressive strength [45].

Dry-stored and dry-tested ProRoot MTA has a compressive strength of 27 MPa [51], while the reported compressive strengths of MTA cements stored in water have reached 86.2 MPa [38]. iRoot FS and iRoot BP, when stored using an accelerated setting method in a hot water bath, have produced compressive strengths of 96 MPa and 177 MPa, respectively [45].

There are no known published results using the ISO 9917.1 test for MTA sealers.

#### 3.2.5. Acid Erosion (ISO 9917)

Lactic acid and sodium lactate are added to water to create a demineralizing solution with pH of 2.74. Samples are placed into specimen holders and given 24 h to set, then removed and immersed in the acidic solution for 24 h. The depth of erosion is measured [17].

There are no known published results using the ISO 9917 test for acid erosion for either MTA restoratives or MTA sealers.

#### 3.2.6. Acid-Soluble Arsenic and Lead Contents (ISO 9917)

Cements are mixed and set for 24 h, then crushed into a powder. Two grams of the powdered cement is then added to 50 mL of HCl, and allowed to stand for 16 h. The solution is then measured for the amount of free arsenic and lead. The maximum permitted content for arsenic and lead is 2 mg/kg and 100 mg/kg, respectively [17].

One study found that both ProRoot MTA and MTA Angelus had levels of arsenic higher than the safe limit specified by ISO 9917 [52]. This result was in contrast to another study that reported both cements as having safe limits of arsenic [53]. Yet another study found that both ProRoot MTA and Ortho MTA (BioMTA, Seoul, Korea) had safe levels of arsenic [54]. Several studies have reported safe levels of lead in MTA cements [52,54].

There are no known published results for MTA sealers.

#### 3.2.7. Genotoxicity and Carcinogenicity (ISO 10993-3)

Genotoxicity testing is a series of in vitro and, under some circumstances, in vivo tests involving assessment of gene mutations in bacteria, chromosomal damage in mammalian cells, the mouse lymphoma tk assay and the mammalian cell micronucleus test for chromosomal damage. In vivo tests can include analysis of bone marrow cells or micronuclei in bone marrow or peripheral blood erythrocytes. For carcinogenicity, materials are implanted into tissues and assessed for tumor development [20].

Numerous tests unanimously show that ProRoot MTA and MTA Angelus cause little or no DNA damage [55,56,57]. MTA Fillapex has shown greater genotoxicity than MTA Angelus [58]. MTA sealers have been modified from the original formulation to alter their handling properties, by adding in various organic (carbon-based) substances. Therefore, MTA sealers should not be assumed to give identical biological responses to the original formulation of MTA.

#### 3.2.8. Cytotoxicity (ISO 10993-5)

The agar diffusion test is a qualitative assessment of cytotoxicity involving culture medium containing serum with melted agar that is compatible with mammalian cells. The specimen is then placed in contact with one-tenth of the cell layer surface, and cytotoxicity is determined from the cellular response after 24–72 h.

A study by Miranda [59] using a 5-point cytotoxicity grading system found that ProRoot MTA and Angelus WMTA received grade 1 (slight cytotoxicity).

Colorimetric assays measure the activity of enzymes that reduce MTT or similar dyes (XTT, MTS, WSTs) to formazan dyes, giving a purple color. These assays allow assessment of cell viability and proliferation in cell culture assays, which provide information on whether a material is cytotoxic. Cell viability can be compared against a negative control (a material which does not produce a cytotoxic response).

ProRoot MTA that has been freshly mixed, i.e., mixed within 1–12 h, shows cytotoxicity (~50% cell survival) [60,61], while samples of ProRoot MTA and MTA Angelus that have set for 1 day or longer consistently show near 100% cell survival [56,62,63,64,65,66].

Samples of iRoot FS and iRoot BP Plus, when given 1 week to set [62,63,67], or even 1 day to set [68], show negligible cytotoxicity, and in this regard, are equivalent to ProRoot MTA. However, fresh samples of iRoot FS show significantly more cytotoxicity than iRoot BP Plus and ProRoot MTA [62]. Furthermore, if iRoot BP Plus is compared against ProRoot MTA in an immediate placement model, iRoot BP Plus gives greater cytotoxicity [69].

MTA sealers show greater toxicity than AH Plus [70]. In a four-week study, MTA Fillapex showed continual cytotoxicity [71,72]. Similarly, iRoot SP showed continual cytotoxicity in a six-week study [36]. Both MTA sealers were more cytotoxic than AH Plus over the same testing period [36,71].

#### 3.2.9. Implantation in Subcutaneous and Intraosseous Tissues (ISO 10993-6)

Implantation studies involve placing materials under the skin of rats and in their jaws and then assessing histologically the appearance of the tissues around the material at different points in time [22].

ProRoot MTA and MTA Angelus cause initial inflammation, which then subsides over a 30- to 90-day period [73,74,75,76].

iRoot FS implanted into subcutaneous tissues is more irritating than ProRoot MTA at 1 week and at 3 weeks [77]. In contrast, iRoot BP Plus is less inflammatory than ProRoot MTA [78].

For MTA sealers, there is no significant difference between iRoot SP and AH Plus [79]. iRoot SP causes less severe subcutaneous connective tissue reactions than MTA Fillapex, but more than a conventional MTA cement [80,81]. The MTA sealer Endo CPM Sealer (EGEO SRL, Buenos Aires, Argentina) causes similar reactions to AH Plus, and similar reactions to MTA cements [75].

When placed into bone, the initial inflammation elicited by MTA decreases over time [74,82,83,84,85,86,87]. The trend seen in studies of this type is that there is moderate inflammation at 7 days, mild inflammation at 30 days, and no inflammation from 60 days onward.

For placement into bone, the MTA sealers MTA Fillapex showed comparable reactions to AH Plus over 28 days [88]. Reactions to iRoot SP were not significantly different to MTA and AH Plus over 60 days [79].

## 4. Discussion

### 4.1. Properties After Mixing

#### 4.1.1. Flow (ISO 6876)

For the ISO test to have an acceptable result, the glass plate above the sealer needs to apply force evenly so that the material shape remains a circle. The operator performing the test is required to balance the glass plate so that the flowing material remains in a circle. The test result is therefore directly affected by the skill and experience of the operator and is subject to bias.

The purpose of the delay of 180 s is possibly an attempt to match the time between when the sealer is mixed and used in clinical practice, where the sealer is dispensed upon a mixing pad and then used to coat gutta percha points. However, some sealers are now dispensed using a syringe, the tip of which can be applied within the root canal, which removes any delay. Some newer sealers are water-based, and exposure to air can cause desiccation, resulting in a reduced flow. With such materials, a delay of 180 s is not appropriate.

In clinical practice, sealers can be applied in a multitude of ways, including injection by syringe as mentioned above, direct application into the root canal using spiral rotary instruments, and by the manual manipulation of gutta percha points on the bench [89]. Each method applies different types and amounts of stress on the sealer which, in turn, alters its viscosity. Sealers are often shear thinning (pseudo-plastic), and show reduced viscosity when the shear rate (i.e., the velocity of the sealer against substrates) is increased [35].

Rather than measuring how far a material flows under constant pressure over many minutes, changes in its viscosity can be measured with rheometers that apply specific shear strains to the material. This method has greater precision than the ISO flow test and thus is more likely to identify significant differences between samples [35]. Furthermore, the application of different shear rates which affects the viscosities has clinical implications. Higher shear rates will lead to lower viscosity of a material and hence increase its ability adapt into voids. However, a low viscosity may also increase the likelihood of periapical extrusion. In this case, rapid placement under excessive pressure would increase the probability of periapical extrusion [35].

When viscosity has been measured at three different shear strains, under all three testing conditions AH Plus had a lower viscosity than MTA Fillapex, which in turn had a lower viscosity than iRoot SP [35]. The advantage of using a rheometer to assess endodontic sealers is that this instrument can measure the viscosity of the material as well as its other important properties such as the viscoelastic moduli (storage and loss moduli), recording how these change over time [90]. Furthermore, a strong negative correlation exists between flow using the glass plate press method, and viscosity, whereby the greater the flow, the lower the viscosity [90]. This is typical of shear thinning behavior.

#### 4.1.2. Film Thickness (ISO 6876)

The ISO test implies that under a particular load, the material should flow in a certain way to produce a specific thickness. However, it does not give guidance as to how a material flows under different loads. This is relevant to endodontics as shear thinning (pseudo-plastic) materials will become less viscous under increasing loads (which lead to higher shear rates), while shear thickening (dilatant) materials will show an increase in viscosity under increasing loads. Endodontic sealers typically exhibit pseudo-plastic properties [35]. Therefore, clinicians can apply excess pressure to intentionally force the material to flow into areas that are more difficult to reach. However, Portland cement, and hence MTA cements in general, typically exhibit shear thickening behavior unless substantial admixtures are present [91]. MTA cements without such additives will not reliably flow into these limited access areas when increased pressure is applied [92].

Measuring the shear strain stress versus shear rate can identify whether a material has pseudo-plastic, dilatant, plastic or Newtonian properties. This provides greater clinical information as to which materials flow better when extra pressure is applied [35]. As discussed above, using rheology the viscosity and storage modulus can be measured, particularly as functions of time and temperature [90].

#### 4.1.3. Working Time (ISO 6876)

The reduction in flow to within 10% of its value does not necessarily correlate with clinical usage of the material, nor does it provide any objective data to assess the handling of the material under clinically relevant conditions of temperature and humidity.

Rheological studies can measure viscosity, storage modulus and loss modulus over time. However, further research is required to determine which points in the storage modulus curve could best be defined as the working time [90].

#### 4.1.4. Setting Time (ISO 6876 and 9917-1)

Having two different standards that can both be used for MTA restoratives results in inappropriate comparisons between products because different methods have been used for assessing their setting times [27]. Setting cements are either defined as set or not set. This dichotomy provides no insight on the transition from setting to set [93]. Both ISO standards involve subjective visual assessment as to whether a material has been completely indented. Furthermore, both standards involve gently placing an indentation needle onto setting cements, a technique that is open to operator variation.

Some MTA restoratives and MTA sealers are not mixed with water, and would not set using ISO 9917.1. Therefore, they are often tested using ISO 6876, relying on water from the damp dental plaster [16]. Set dental plaster is a porous material filled with water, with the amount of water depending upon the mixing ratio of powder to water [94]. The rate of diffusion of water from the porosities of the dental plaster mold may vary between samples as well as from day to day according to how the plaster has been prepared. To increase the water available in the plaster mold, it can be intentionally submerged in water prior to placement of the cement, followed by placement of the cement while submerged in water. When tested with this altered methodology iRoot^®^ FS (Innovative BioCeramix, Vancouver, Canada) and iRoot BP gave settings time of 18 min and 62 min, respectively [45].

Despite the perceived faster setting time of iRoot BP compared to MTA, when these materials are contaminated with blood, the settings reactions are adversely affected. MTA requires 36 h to set, while iRoot BP is not set even 48 h after mixing [95]. These findings are in stark contrast to the advertised setting times, which are 4 h for ProRoot MTA and 2 h for iRoot BP. Therefore, the methodology of curing iRoot BP in the laboratory where moisture is added from plaster is clinically inaccurate, since MTA is often placed against blood. As well, studies utilizing gypsum models, such as ISO 6876, suffer from variations from the plaster water content, unlike samples tested in metal molds.

The definitions for initial set and final set, which are similar to those in ISO 6876 and ISO 9917 respectively, can be confusing as they provide little clarification as to whether a material has set. In the case of iRoot SP, the initial setting time will be retarded and the final setting time accelerated by the presence of water [36].

Other studies have used rheology to define the setting time. Through the use of storage modulus, the setting time of MTA can be defined as the point in time when the modulus reaches 8 × 10^8^ Pa [96] or 95% of the plateau storage modulus [93]. Storage modulus has been considered useful for assessing the setting time of endodontic sealers [90]. However, the time to reach a defined value or percentile of storage modulus is not used widely as a means to determine setting time.

For testing purposes, materials that require passive diffusion of moisture to cause the material to set can utilize humid chambers. Alternatively, the system can be submerged during testing. When cements that do not require passive diffusion of moisture to set are to be compared against cements that do require passive diffusion of moisture to set, all should be tested under the same conditions.

### 4.2. Properties After Setting:

#### 4.2.1. Dimensional Change (ISO 6876)

Although iRoot SP is a hygroscopic cement and will require water for its setting reaction, varying the test methods used with iRoot SP when it is compared to other cements reduces the validity of the results. Both MTA Fillapex and AH Plus absorb some water [97]. If these were tested in the same modified way as iRoot SP, their values for dimensional change would likely change. When MTA cements absorb water, they expand [98]. When samples are tested, they should not be submerged in water but rather in a buffered saline solution so that there are physiological concentrations of ions, and the testing conditions are more aligned to clinical conditions [40].

#### 4.2.2. Solubility (ISO 6876)

MTA is more soluble in distilled water than in isotonic solutions. The ISO test uses hypotonic solutions, which increases the solubility far beyond that in the clinical situation. When MTA is tested in physiological solution, Hank’s balanced salt solution and phosphate buffered solution, BioAggregate, Biodentine and ProRoot MTA illustrated negative values indicating that they had absorbed ions from the environment, rather than an overall loss of mass [23,26]. Hence, the clinical relevance of this method of testing is questionable [99].

The test also involves giving cements the opportunity to set for a period of 50% longer than the setting time stated by the manufacturer. Those setting times often utilize ISO 6876, and thus may not reflect whether this material has reached its final hardness. As an example, ProRoot MTA when tested under ISO 6876 has a setting time of 78 min, but when tested under ISO 9917.1 has a setting time of 4 h [27,33]. Allowing a period of 50% may be insufficient if the goal is to test completely set samples.

Once fully set, MTA contains calcium hydroxide and can produce some alkalinity after 28 days [100]. On this basis, it could be argued that if a completely set material is desired, testing the cement should occur after it has aged at least 28 days. For clinical relevance, solubility should be tested immediately after placement. This could involve a slow submergence, at some arbitrary rate, preferably into a physiological solution or into blood.

#### 4.2.3. Radiopacity (ISO 6876 and 9917-1)

There is a wide range of reported values for radiopacity within various cements and some issues around the details of the method for determining radiopacity [101]. Minor variations in exposure time, or in target distance within the allowed range of 300–400 mm do not significantly influence the results [102]. One could expect minor variations according to the level of curing between samples [103] and the type of imaging system used [104].

The ISO standards describe the use of optical density instruments on exposed films [16,17]. Digital imaging enables objective quantification with greater specificity, through the use of grayscale histogram analysis of greyscales of radiopacity [105,106].

Regardless of the methods used, it seems the most meaningful comparisons are tests that involve several cements within the one study so that direct comparisons can be made between materials, rather than comparisons between studies where more variables are at play [105].

Alternatively, a clinically relevant test based on the clinical appearance and usage of MTA in radiographs could be considered [106], possibly in a tissue simulator model with teeth and bone [107]. Solubility affects radiopacity with more soluble MTAs illustrating greater loss of radiopacity over time [23]. Future studies should assess changes in radiopacity as dissolution occurs.

#### 4.2.4. Compressive Strength (ISO 9917)

MTA will expand when stored fully immersed in physiological solutions but will shrink when stored in a humid chamber [40]. Therefore, it is important to know whether samples are stored dry, stored in a humid chamber, or immersed completely in water or other fluids [51,108].

For assessing compressive strength, most studies in the literature test samples of MTA once the cement has been allowed to age for 21–28 days prior to testing [43,44,45], which is a major difference from what is allowed in ISO 9917 [17].

Ideally, MTA should be wet cured, i.e., submerged in a physiological buffer solution, to prevent desiccation of samples, and tested in physiological solution (e.g., phosphate buffered saline), to replicate physiological conditions [109]. While there is logic in testing samples that have aged for 304 weeks, there is also value in testing samples at earlier points in time, e.g., 1 day, 1 week and 3 weeks, to track how compressive strength develops over time. At any given point in time after mixing, if a cement has not set, it will have no compressive strength. This information is of value to the clinician. A further point of clinical relevance is exposure to blood. MTA that has been cured in the presence of blood has reduced compressive strength [110].

#### 4.2.5. Acid Erosion (ISO 9917)

This test is suitable for dental restoratives which are exposed to the acids in the oral environment. MTA, whether it be used as a restorative material or as a sealer, is typically located under coronal restorations, and not exposed to the oral environment. The risk of acid erosion from exposure to lactic acid produced by dental plaque biofilm is not a clinically relevant risk.

Set MTA has increased solubility in acidic environments, and these conditions may occur wherever inflammation is present [111]. As with solubility studies, a more clinically relevant method would be to test samples that have been exposed to weak acids immediately after mixing, rather than testing the material once it has cured fully under neutral pH conditions.

#### 4.2.6. Acid-Soluble Arsenic and Lead Contents (ISO 9917)

The results of the ISO test for acid-soluble arsenic and lead content show inconsistent patterns of results. This may be due to differences in types of acids and concentrations used, as well as variations in exposure time to those acids [54]. Variations in the results can also be caused by how the samples are prepared by grinding them into powder using a mortar and pestle. Materials of different hardness will be ground to different fineness, and hence will have a different surface area. This will affect how much heavy metals can be leached out from exposure to acid. The clinical relevance of this test is questionable since MTA is not exposed to the occlusion where it can undergo attrition, or be exposed to strong acids. Biological response tests, such as implantation studies, are of greater clinical relevance. There is no direct relationship between the concentration of arsenic found in MTA and the associated inflammatory response in the tissues [112].

#### 4.2.7. Genotoxicity and Carcinogenicity (ISO 10993-3)

The current panel of tests which use bacterial and mammalian cells in culture may not represent what happens in human tissues. Implantation tests in animals are more relevant, but may not be practicable for assessing long-term safety issues such as carcinogenicity.

#### 4.2.8. Cytotoxicity (ISO 10993-5)

The cytotoxicity of MTA cement is affected by several variables, including storage time and storage media. The key consideration is the release of calcium hydroxide from the cement as it is setting. Initially, the speed of the setting reaction is high, and cytotoxic effects are seen from released calcium hydroxide. As the material reaches its final set, much of the alkalinity is lost and hence there is less cytotoxicity [100]. Therefore, any test method where the MTA sample is fully set and then is placed into a culture or against tissues is not reflective of the clinical use of the material. On the other hand, putting freshly mixed MTA cement directly into a cell culture well will likely result in the disintegration of the material, giving greater alkaline effects from the calcium oxide components of the Portland cement. Similar considerations would apply to sealers, but with the caveat that the viscosity modifiers used in these could also affect cell viability [72]. An alternative method would be to place freshly mixed MTA immediately into a simulated root end filling that is then exposed to the testing culture [69]. This prevents the disintegration of the MTA.

Cytotoxicity tests employ short testing periods of typically 1–3 days. This will not identify concerns of sustained toxicity due to slow dissolution or degradation over time. This is especially important for MTA sealers which have high solubility [72]. These materials should be tested over longer periods of time. Samples can be prepared, placed into a simulated root end filling, allowed to cure in water for 1, 2 or 3 weeks, and then placed into cell culture.

#### 4.2.9. Implantation in Subcutaneous and Intraosseous Tissues (ISO 10993-6)

Results from subcutaneous implantation are considered of less relevance than those from intraosseous implantation since the clinical usage of MTA is within osseous structures.

When interpreting the results of inflammation in implantation studies, it is important to understand that, as stated by Sumer [73], “when assessing the biocompatibility of a material, later harmful effects are considered to be more important than its initial effects.” The initial inflammation is partly due to the trauma of the surgical procedure to implant the material, and partly due to the material itself. Review periods should account for this, and use longer intervals such as 7 days, 30 and 60 days.

Although animal biocompatibility studies are considered superior to cell culture studies, it must be remembered that a material which appears to be well tolerated and which does not elicit intense inflammation may have inferior physical properties, such as high solubility or shrinkage, which compromise its performance.

Studies using larger animals, e.g., beagle dogs, where MTA is placed into root end fillings in a location where apical periodontitis has been induced, provide a method of assessing osseous healing. This is an important consideration as it goes beyond the inflammatory response. Furthermore, the use of larger animals in this manner enables not only histological assessment as per ISO 10993-6 but also the radiographic assessment of healing. However, large animal welfare can prohibit such studies being undertaken [113].

## 5. Conclusions

MTAs can be separated into two main types, MTA restoratives and MTA sealers, and feature products which are often marketed as bioceramics. These endodontic bioceramics, as with MTA, fall under the categories of MTA restoratives and MTA sealers.

The results for ISO tests used for testing MTA can be biased by curing method of the MTA. MTA should be cured in a way that represents the clinical usage of the material. This typically involves immediate placement and immediate testing of samples rather than curing the cement outside of testing conditions.

## Figures and Tables

**Table 1:** Elemental composition comparisons of popular MTA (Mineral trioxide aggregate) restorative brands.

MTA Restoratives	ProRoot MTA ^1^ [9]	MTA Angelus ^2^ [10]	Biodentine [10]	Endocem MTA ^3^ [9]	Endocem Zr ^3^ [9]	Bioaggregate [11]
Calcium	37.18	43.4	45.3	40.21	18.52	38.5
Silicon	11.17	7.60	9.2	10.2	4.82	11.5
Oxygen	32.98	34.20	41.7	15.34	18.52	37.4
Sulfur	1.11	-	-	1.99	0.86	-
Aluminium	0.93	1.50	-	3.33	1.45	-
Phosphorus	-	-	-	-	-	1.90
Bismuth	15.75	12.8	-	24.24	-	-
Zirconium	-	-	3.5	-	57.49	-
Tantalum	-	-	-	-	-	10.6

^1^ Dentsply Sirona, York, USA; ^2^ Angelus, Londrina, Brazil; ^3^ Maruchi, Wonju-si, South Korea

**Table 2:** Composition comparisons of MTA sealer brands.

MTA Sealers	Composition
BioRoot RCS [15]	Powder: Tricalcium silicate, povidone, zirconium oxide. Liquid: Aqueous solution of calcium chloride and polycarboxylate.
EndoSeal MTA [14]	Paste: Calcium silicates, calcium aluminates, calcium aluminoferrite, calcium sulfates, radiopacifier, thickening agents.
iRoot SP [14]	Paste: Calcium silicates, calcium phosphate monobasic, calcium hydroxide, filler, zirconium oxide, thickening agents.
MTA Fillapex [13]	Paste 1: salicylate resin, fumed silica, and bismuth trioxide as the radiopaque agent). Paste 2: MTA (40%), fumed silica, titanium dioxide, and 1,3-butylene glycol disalicylate resin.

**Table 3:** Properties of MTA restoratives and sealers after mixing.

Commercial Products	ISO 6876Flow (mm)	ISO 6876 Film Thickness (µm)	ISO 6876 Working Time (min)	ISO 6876 Setting Time (min)	ISO 9917.1 Setting Time (min)
*MTA Restoratives*					
BioAggregate	-	-	-	-	1260 [23]
Biodentine	6.5 [24] ^1^	-	-	6.5 [25]	45–85.7 [23,26]
EndoCem MTA	-	-	-	4 [27]	78 [27]
MTA Angelus	6.3–13.6 [24,28] ^1^	101 [28] ^2^	-	8.5–24.3 [25,29,30]	171–175 [29,30]
ProRoot MTA	14.2 [31] ^1^	-	6 [31]	2.5–165 [27,31,32]	140–284 [26,27,32,33]
*MTA Sealers*					
BioRoot RCS	16 [34] ^1^	52 [34] ^2^	-	-	-
EndoSeal MTA	20.2 [14]	-	-	-	-
iRoot SP	23.1 [35]	22 [35]	>1440 [35]	162 [35] ^3^ or 4320–6480 [36] ^4^	10,080–14,400 [36] ^4^
MTA Fillapex	24.9 [35]	23.9 [35]	45 [35]	66 [35]	-
Epoxy resin control	-	-	-	-	-
AH Plus	17–21.2 [34,35]	15–16 [34,35]	240 [35]	690 [35]	-

**^1^** Fails ISO 6876 standard for a minimum of 17 mm [16]. ^2^ Fails ISO 6876 standard for a minimum of no greater than 50 µm [16]. ^3^ Performed using accelerated setting conditions [35]. ^4^ As greater amounts of water (0–9%) are provided for the setting of iRoot SP, the initial setting time increases from 72 h to 108 h, while the final setting time decreases from 168 h to 240 h [36].

**Table 4:** Non-biological properties of MTA restoratives and sealers after setting.

Commercial Products	ISO 6876 Dimensional Change (%)	ISO 6876 Solubility (%)	Radiopacifier(s)	ISO 6876 and ISO 9917.1 Radiopacity in mm Al	ISO 991.1 Compressive Strength (MPa)
*MTA Restoratives*					
Bioaggregate	-	-	Ta_2_O_5_	5.0–5.7 [23] ^5^	16.34–29.07 [23,38]
Biodentine	-	4.61 [26] ^2^	ZrO_2_	1.5–2.8 [26,39] ^4^, 3.3–4.1 [23] ^5^	67.18–170.8 [23,25]
MTA Angelus	-	0.82–3.7 [29,30,40] ^2^	Bi_2_O_3_	4.5–5.96 [28,29,30,39]	19.63–41.51 [25,29]
NeoMTA	-	-	Ta_2_O_5_	3.8 [41]	
ProRoot MTA	0.30 [32]	1.1–1.5 [26,32,40]	Bi_2_O_3_	6.4–8.5 [26,32,42]	27 [32] 65–86.23 [38,43,44] ^6^
iRoot BP	-	-	-	-	177 [45] ^7^
iRoot FS	-	-	-	-	96 [45] ^7^
*MTA Sealers*					
BioRoot RCS	-	-	ZrO_2_	8.3 [34]	-
EndoSeal MTA	0.21 [14]		ZrO_2_	9.50 [14]	-
iRoot SP	0.087 [35] ^1^	2.9 [35] ^3^ 20.64 [46] ^2^	ZrO_2_	3.0–6.68 [14,47]	-
MTA Fillapex	–0.67 [35]	1.1 [35] ^3^ 5.65–14.89 [28,46] ^2^	Bi_2_O_3_	6.5–9.4 [28,48]	-
Epoxy resin control	-	-	-	-	-
AH Plus	–0.034 [35]	0.06–0.28 [35,46]	CaWO_4_, ZrO_2_	6.9–18.4 [14,34,47,48]	22 [49]

**^1^** In this study, the ISO test was modified to provide extra water to enable a complete set of iRoot SP [35]. ^2^ Fails ISO 6876 standard of a maximum of 3% [16]. ^3^ The solubility test was modified by submerging the molds into heated water, hence providing more water to enable the complete setting. ^4^ Fails ISO 6876 standard of a minimum of 3 mm Al [16]. ^5^ Radiopacity was tested at day 1 and day 28 of immersion in Hank’s balanced salt solution [23]. ^6^ These samples were cured in wet conditions rather than dry conditions. ^7^ In this study, the ISO test was modified using an accelerated setting method in a hot water bath [45].
